# Results from a Phase 1b/2 Study of Ibrutinib Combination Therapy in Advanced Urothelial Carcinoma

**DOI:** 10.3390/cancers15112978

**Published:** 2023-05-30

**Authors:** Nataliya Mar, Yousef Zakharia, Alejandro Falcon, Rafael Morales-Barrera, Begona Mellado, Ignacio Duran, Do-Youn Oh, Stephen K. Williamson, Pablo Gajate, Hendrik-Tobias Arkenau, Robert J. Jones, Min Yuen Teo, Tolga Turan, Robert T. McLaughlin, Hillary M. Peltier, Elizabeth Chong, Harisha Atluri, James P. Dean, Daniel Castellano

**Affiliations:** 1 Division of Hematology/Oncology, University of California Irvine, Orange, CA 92868, USA; 2 Division of Hematology/Oncology, University of Iowa Hospitals and Clinics, Iowa City, IA 52242, USA; 3 Hospital Universitario Virgen del Rocío, 41013 Seville, Spain; 4 Vall d’Hebron Institute of Oncology, Vall d’Hebron University Hospital, Universitat Autònoma de Barcelona, 08035 Barcelona, Spain; 5 Medical Oncology Department, Hospital Clínic i Provincial de Barcelona, Institut D’Investigacions Biomediques August Pi i Sunyer (IDIBAPS), University of Barcelona, 08036 Barcelona, Spain; 6 Hospital Universitario Marqués de Valdecilla, Instituto de Investigación Valdecilla (IDIVAL), 39011 Santander, Spain; 7 Seoul National University Hospital, Cancer Research Institute, Seoul National University College of Medicine, Seoul National University Graduate School, Seoul 03080, Republic of Korea; 8 University of Kansas Hospital Cancer Center, Kansas City, KS 64114, USA; 9 Hospital Universitario Ramón y Cajal, 28034 Madrid, Spain; 10 Sarah Cannon Research Institute United Kingdom (SCRI-UK) and University College London Cancer Institute, London W1G 6AD, UK; 11 Beatson West of Scotland Cancer Centre, University of Glasgow, Glasgow G12 0YN, UK; 12 Genitourinary Oncology Service, Department of Medicine, Memorial Sloan Kettering Cancer Center, New York, NY 10065, USA; 13 Pharmacyclics LLC, an AbbVie Company, South San Francisco, CA 94080, USA; 14 Hospital Universitario 12 de Octubre, 28041 Madrid, Spain

**Keywords:** urothelial carcinoma, ibrutinib, paclitaxel, pembrolizumab

## Abstract

**Simple Summary:**

Urothelial carcinoma (UC) is a common type of bladder cancer. Patients with UC that has not improved after previous treatment or has spread to other parts of the body need new treatment options. Ibrutinib is a drug approved for the treatment of blood cancers and chronic graft-versus-host disease. It works by blocking Bruton’s tyrosine kinase, an enzyme important for cancer cell survival. Other drugs, pembrolizumab and paclitaxel, are considered standard treatments for UC. This study aimed to understand if ibrutinib alone or combined with pembrolizumab or paclitaxel could lessen disease in patients with UC and help them live longer without their disease getting worse. More patients previously treated for UC had disease improvement with the combinations of ibrutinib and pembrolizumab or ibrutinib and paclitaxel than with pembrolizumab, paclitaxel or ibrutinib alone or compared to historical data. Based on these results, additional studies of ibrutinib combinations in UC are needed.

**Abstract:**

Ibrutinib is a first-in-class Bruton’s tyrosine kinase inhibitor approved for the treatment of various B-cell malignancies and chronic graft-versus-host disease. We evaluated the safety and efficacy of ibrutinib, alone or combined with standard-of-care regimens, in adults with advanced urothelial carcinoma (UC). Once-daily ibrutinib was administered orally at 840 mg (single-agent or with paclitaxel) or at 560 mg (with pembrolizumab). Phase 1b determined the recommended phase 2 dose (RP2D) of ibrutinib, and phase 2 assessed progression-free survival (PFS), overall response rate (ORR), and safety. Thirty-five, eighteen, and fifty-nine patients received ibrutinib, ibrutinib plus pembrolizumab, and ibrutinib plus paclitaxel at the RP2D, respectively. Safety profiles were consistent with those of the individual agents. The best-confirmed ORRs were 7% (two partial responses) with single-agent ibrutinib and 36% (five partial responses) with ibrutinib plus pembrolizumab. Median PFS was 4.1 months (range, 1.0–37.4+) with ibrutinib plus paclitaxel. The best-confirmed ORR was 26% (two complete responses). In previously treated patients with UC, ORR was higher with ibrutinib plus pembrolizumab than with either agent alone (historical data in the intent-to-treat population). ORR with ibrutinib plus paclitaxel was greater than historical values for single-agent paclitaxel or ibrutinib. These data warrant further evaluation of ibrutinib combinations in UC.

## 1. Introduction

Bladder cancer accounted for approximately 573,000 new cases and more than 210,000 deaths worldwide in 2020 [1], with about 90% of cases arising from urothelial cells [2]. Advanced urothelial carcinoma (UC) has a poor prognosis, with patients surviving a median of 9 to 18 months [3,4,5]. Standards of care for advanced UC continue to improve, as seen with the addition of maintenance avelumab to best supportive care after first-line chemotherapy, which demonstrated a significantly prolonged overall survival (OS) (median OS 21.4 months vs. 14.3 months with best supportive care alone) [6]. However, treatments with more durable responses, as well as chemotherapy-free treatment options, are needed.

Ibrutinib is a first-in-class, once-daily covalent inhibitor of Bruton’s tyrosine kinase (BTK) approved for the treatment of various B-cell malignancies and chronic graft-versus-host disease following the failure of 1 or more lines of systemic therapy [7]. Ibrutinib targets the B-cell receptor pathway and immune cells in the tumor microenvironment via BTK, as well as kinases relevant to solid tumor treatment [8,9,10], including tyrosine kinase ETK/BMX [11] and interleukin-2–inducible T-cell kinase (ITK) [12]. Ibrutinib inhibits ITK under physiologic conditions, potentially shifting helper T-cell polarization to a phenotype with a stronger antitumor activity [12]. Further, the disruption of BTK signaling may modify the microenvironment of solid tumors, thus increasing their drug sensitivity. Therefore, combining ibrutinib with standard-of-care or mechanistically complementary agents may boost antitumor activity and improve outcomes in patients with advanced UC.

This phase 1b/2 clinical study was conducted to explore the safety, tolerability, and preliminary activity of ibrutinib, administered as a single agent or in combination with paclitaxel or pembrolizumab, in previously treated patients with advanced UC who had progressed on 1 or more lines of therapy. The results from other study cohorts conducted in patients with renal cell carcinoma, gastric adenocarcinoma, or colorectal adenocarcinoma are reported separately (submitted publication).

## 2. Materials and Methods

### 2.1. Study Design and Patients

This open-label, phase 1b/2 multicenter study (ClinicalTrials.gov; NCT02599324) was conducted between December 2015 and August 2021 in 3 cohorts of previously treated patients with UC. The primary objective of phase 1b was to determine the recommended phase 2 dose (RP2D) of ibrutinib administered orally as a single agent or in combination with paclitaxel or pembrolizumab. The primary objectives of phase 2 were to assess the overall response rate (ORR) of single-agent ibrutinib or ibrutinib plus pembrolizumab and the progression-free survival (PFS) of ibrutinib in combination with paclitaxel. Secondary objectives in the phase 2 portion included assessment of PFS with ibrutinib alone or with pembrolizumab, ORR with ibrutinib plus paclitaxel, and the following in all cohorts: disease control rate (DCR), duration of response (DOR), OS, safety, and tolerability. Exploratory objectives included the evaluation of pharmacokinetics in each cohort. The data cut-off date for this analysis was 4 October 2021.

For patients receiving single-agent ibrutinib, the RP2D of 840 mg per day was confirmed in the first 6 evaluable patients. In phase 2, at least 13 patients were enrolled into stage 1 of the Simon’s 2-stage design, including any from phase 1b who were treated at the RP2D.

For patients receiving ibrutinib plus pembrolizumab, phase 1b followed a 6 + 3 dose de-escalation design to evaluate dose-limiting toxicities (DLTs) and determine the RP2D. A 3 + 3 + 3 design was used for patients receiving ibrutinib plus paclitaxel. Details regarding study design and DLT assessment are included in the statistical considerations and analysis populations section.

In phase 2, the 2 combination therapy cohorts received ibrutinib at the RP2D determined in phase 1b in combination with intravenous (IV) pembrolizumab 200 mg every 3 weeks or weekly IV paclitaxel 80 mg/m^2^, respectively, until disease progression or unacceptable toxicity. Following the determination of the RP2D by a dose level review committee, additional patients were enrolled and treated in phase 2 at the RP2D to further evaluate the efficacy of the regimen for each cohort as prespecified. In the single-agent cohort, ibrutinib was orally administered daily at the RP2D in 21-day cycles until disease progression or unacceptable toxicity. All patients were aged ≥18 years with adequate hematologic, hepatic, and renal function and an Eastern Cooperative Oncology Group (ECOG) performance status score of 0 or 1. To be eligible for the single-agent ibrutinib cohort, patients must have received between 1 and 2 prior lines of therapy, 1 of which must have included a checkpoint inhibitor. Eligible patients receiving ibrutinib plus pembrolizumab had either locally advanced or metastatic UC (if without prior treatment, were not eligible for cisplatin chemotherapy and had a programmed death ligand 1 [PD-L1] score of ≥10; if previously treated, had received 1 to 2 prior regimens, and had progressed on platinum chemotherapy or were within 12 months of neoadjuvant or adjuvant platinum chemotherapy). To be eligible for the ibrutinib plus paclitaxel cohort, patients must have received between 1 and 2 prior lines of therapy, including 1 platinum-based regimen.

All patients provided written informed consent for participation in this study, as approved by the Institutional Review Board, Research Ethics Board, and Independent Ethics Committee before any study-specific screening procedures were performed.

### 2.2. Assessments and Analyses

Tumor response was assessed using computed tomography or magnetic resonance imaging. Imaging was performed at baseline and every 6 weeks thereafter, per the investigator using RECIST v1.1 guidelines, including confirmation of complete responses (CRs) and partial responses (PRs) at least 28 days after the criteria for response were first met [13]. The best overall response (BOR) was defined as the best response recorded from the start of treatment until disease progression or recurrence. Additional response definitions are included in the Supplementary Methods. Treatment-emergent adverse events (TEAEs) were graded based on Common Terminology Criteria for Adverse Events version 4.03 [14]. All TEAEs were documented from the time of the first dose of study treatment until 30 days following the last dose for ibrutinib or companion drug (90 days for the pembrolizumab cohort) or the day before initiation of subsequent anticancer treatment, whichever occurred first.

Plasma samples were collected for all patients for pharmacokinetic determination of ibrutinib in all cohorts and of paclitaxel in the combination therapy cohorts, respectively.

Kinase expression levels in baseline tumor biopsies for a small number of patients were assessed using a NanoString gene expression assay (NanoString Technologies, Inc., Seattle, WA, USA). Next-generation sequencing (Personalis, Inc., Menlo Park, CA, USA) was used to evaluate RNA and DNA-seq-based association of individual genes and signatures with response and survival in pre-treatment patient tumor samples. Additional sequencing analysis details are included in Supplementary Methods.

### 2.3. Statistical Considerations and Analysis Populations

For each UC cohort, the study design provided 80% power to perform 1-sided hypothesis testing at an α level of 0.05. The all-treated population for each cohort, defined as patients who received ≥1 dose of study treatment, was used to summarize all data unless otherwise indicated. The efficacy-evaluable population for each cohort was defined as eligible patients who received ≥1 dose of ibrutinib at the RP2D as a single agent or in combination with ≥1 dose of the companion drug. Patients receiving ibrutinib alone or in combination with pembrolizumab had measurable disease (≥1 target lesion at baseline) and had at least 1 adequate post-baseline overall disease assessment per RECIST v1.1 guidelines. Patients receiving ibrutinib plus paclitaxel also had at least 1 post-baseline overall disease assessment per RECIST v1.1 guidelines or died prior to the first adequate post-baseline overall disease assessment. The efficacy evaluable population was used for reporting efficacy data.

For the single-agent ibrutinib cohort, up to 27 patients were planned to be enrolled based on Simon’s 2-stage minimax design to test the null hypothesis of a historical response rate of 5% against the target response rate of 20% (alternative hypothesis) at a 1-sided α level of 0.05; a total of 35 patients were included in the all-treated population. Similarly, for the ibrutinib plus pembrolizumab cohort, up to 53 patients were planned to be enrolled based on Simon’s 2-stage minimax design to test the pembrolizumab response rate of 20% (null hypothesis) against the target response rate of 35% (alternative hypothesis) at a 1-sided α level of 0.05. The pembrolizumab cohort accrual was terminated by the sponsor prior to reaching the stage 1 milestone of the Simon’s 2-stage design, enrolling a total of 18 patients. For ibrutinib plus paclitaxel, a sample size of approximately 55 evaluable patients allowed testing of the null hypothesis that the median PFS was ≤2.3 months versus the alternative hypothesis of PFS ≥4.1 months at a 1-sided α level of 0.05; a total of 59 patients were included in the all-treated population.

Fisher’s exact test, implemented in R version 3.5, was used to test the significance of mutation association with response. An unbiased validation of the differentially mutated subnetwork, including *KDM6A* and *KMT2D*, was conducted using DAMOKLE (Python 2.7) and STRING protein–protein interaction database version 11.5. To visualize the mutation data, oncoprint and mutated gene bar plots were created using Maftools version 2.3.40.

### 2.4. Data Availability

Requests for access to individual participant data from clinical studies conducted by Pharmacyclics LLC, an AbbVie Company, can be submitted through the Yale Open Data Access (YODA) Project site at http://yoda.yale.edu (accessed on 23 May 2023).

## 3. Results

### 3.1. Phase 1b

#### Baseline Characteristics and Patient Disposition

For the single-agent ibrutinib cohort, 10 patients enrolled and received ibrutinib at 840 mg orally daily. The median time on study was 16 months (range, 0.7–17); the median time on treatment was 1.2 months (range, 0.6–3.0). Seventeen patients enrolled and received ibrutinib 560 mg plus pembrolizumab in phase 1b. The median time on study was 10 months (range, 0.5–20.1); the median time on treatment was 2.3 months (range, 0.6–3.0). Fourteen patients enrolled and received ibrutinib plus paclitaxel in the phase 1b cohort; patients received ibrutinib 560 mg (*n* = 4) or ibrutinib 840 mg (*n* = 10) orally daily. The median time on study was not estimable (range, 1.6–44.7); the median time on treatment was 1.6 months (range, 0.4–11.5). No DLTs were reported in any cohort, thereby confirming the RP2Ds of 840 mg of single-agent ibrutinib, 560 mg of ibrutinib plus pembrolizumab, and 840 mg of ibrutinib plus paclitaxel.

### 3.2. Phase 1b/2 at RP2D

#### Baseline Characteristics and Patient Disposition

Baseline patient and disease characteristics and patient disposition data for each cohort are shown in Table 1. The background and representativeness of the study populations are shown in Table S1. In the single-agent ibrutinib cohort, 35 patients (median age, 71 years [range, 52–88]; 74% [26/35] male) enrolled in phase 1b/2. The majority of patients (86%; *n* = 30/35) had received two prior regimens for UC. Thirty-four of 35 patients received prior systemic therapy in the metastatic setting, with the majority having received two regimens (21 patients, 60%). Thirteen patients received prior therapy in the adjuvant and/or neoadjuvant setting; 91.4% of patients received a checkpoint inhibitor in the locally recurrent and/or metastatic setting, and 45.7% received a prior platinum agent in this setting. One patient received prior systemic therapy in the adjuvant and/or neoadjuvant setting only. All 10 patients in phase 1b received the RP2D of ibrutinib 840 mg. The median time on study was 13.5 months (range, 0.7–17.3). The median average daily dose of ibrutinib was 835 mg per day, and the median relative dose intensity of ibrutinib was 99%.

Eighteen patients (median age 70 years [range, 52–84]; 72% [13/18] male; 72% [13/18] received ≥1 prior regimen) were enrolled in the ibrutinib plus pembrolizumab cohort in phase 1b/2; all 18 patients received ibrutinib at 560 mg and pembrolizumab (200 mg IV every 3 weeks). The median time on study was 10.4 months (range, 0.5–20.1). The median average daily dose of ibrutinib was 556 mg per day, and the median relative dose intensity of ibrutinib was 99%. The median cumulative dose of pembrolizumab was 700 mg/m^2^. Three of eighteen patients (17%) experienced a dose hold and dose modification because of an adverse event. Four patients (22%) missed a dose of pembrolizumab for ≥1 cycle. Five of 18 patients were treatment-naive; more than half of the patients (61.1%) received one prior regimen, and two patients (11.1%) received two prior regimens. Seven of eighteen patients received prior systemic treatment in the metastatic setting, with all seven receiving a prior platinum agent. Eight of eighteen patients received prior treatment in the adjuvant and or neoadjuvant setting.

All 59 patients that enrolled in the ibrutinib plus paclitaxel cohort in the phase 1b/2 cohort received the RP2D of ibrutinib at 840 mg and paclitaxel (80 mg/m^2^ IV every week). The median age of patients in this cohort was 68 years (range, 48–90), and 86% (51/59) of patients were male. Twenty-eight patients (47.5%) (28/59) received one prior regimen, and 52.5% (31/59) of patients had received two prior regimens. The majority of patients (55) received prior treatment in the metastatic setting. Thirty of fifty-nine patients (50.8%) received prior adjuvant and/or neoadjuvant therapy. The median time on study was 37.6 months (range, 0.9–44.7). The median average daily dose of ibrutinib was 676 mg per day, the median relative dose intensity of ibrutinib was 81%, and the median time on all study treatments was 2.5 months (range, 0.2–38.0).

### 3.3. Efficacy at RP2D

**Figure 1:**
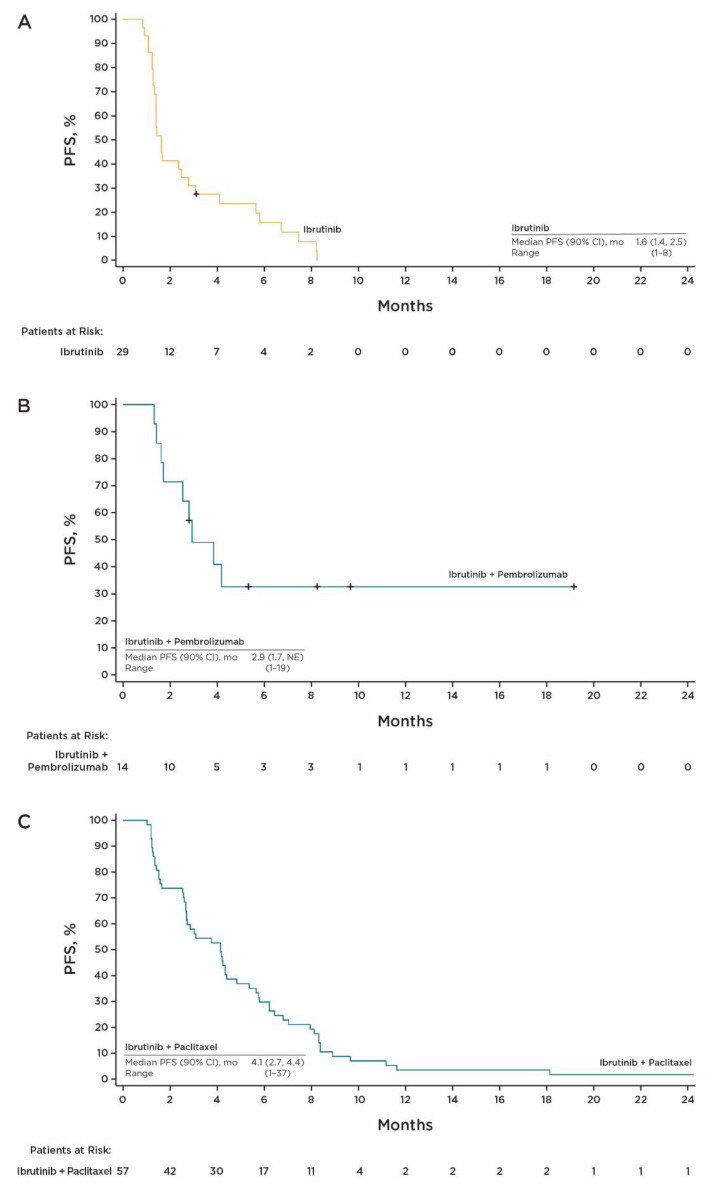
PFS per investigator assessment for (**A**) single-agent ibrutinib, (**B**) ibrutinib plus pembrolizumab, and (**C**) ibrutinib plus paclitaxel. Abbreviations: NE, not evaluable; PFS, progression-free survival.

**Figure 2:**
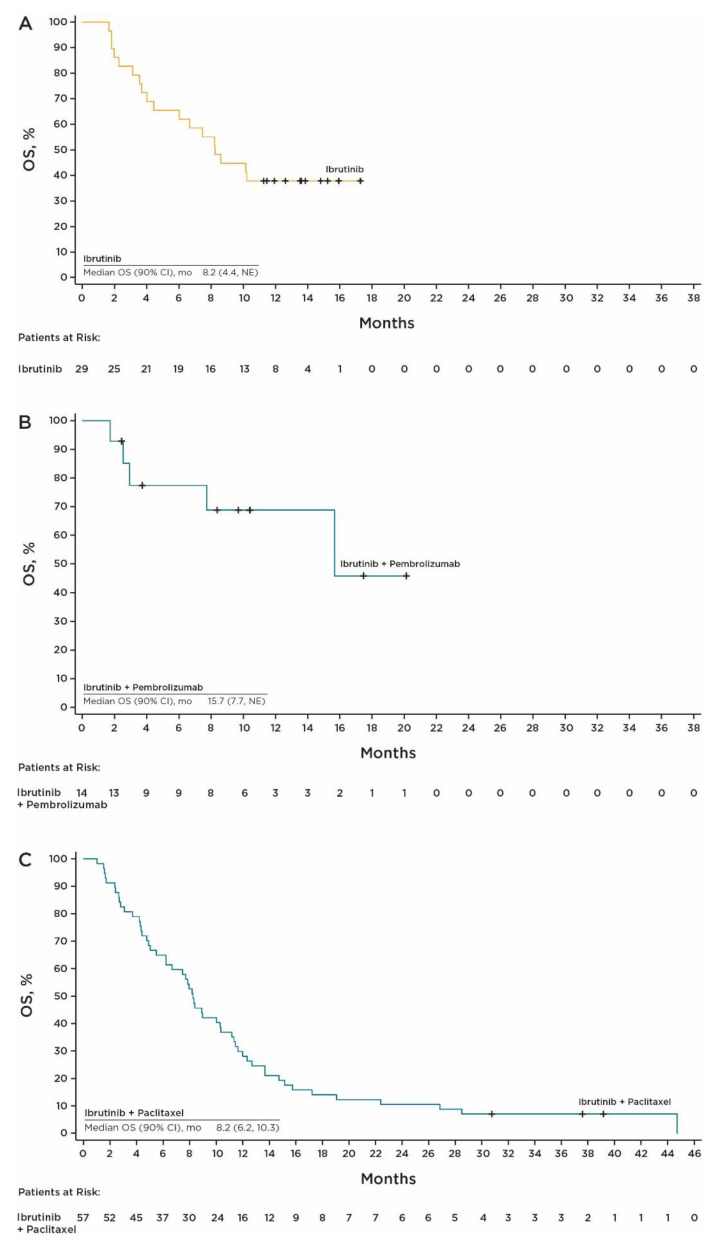
OS with (**A**) single-agent ibrutinib, (**B**) ibrutinib plus pembrolizumab, and (**C**) ibrutinib plus paclitaxel. Abbreviations: NE, not evaluable; OS, overall survival.

With single-agent ibrutinib, the best confirmed ORR was 6.9% (90% CI, 1.2, 20.2), with two PRs. The DCR based on BOR was 48.3% (90% CI, 32.0, 64.8). Median PFS was 1.6 months (range, 0.9–8.2) (Figure 1A). Twenty-eight of twenty-nine efficacy evaluable patients (96.6%) had progressive disease (PD) (*n* = 24) or died (*n* = 4) during the study. The 6-month PFS rate was 15.8% (90% CI, 6.4, 28.8). Among confirmed responders in the efficacy evaluable population, the median DOR was 3.5 months (90% CI, 3.1, not evaluable [NE]). Median OS was 8.2 months (90% CI, 4.4, NE) (Figure 2A).

In the ibrutinib plus pembrolizumab cohort, the best confirmed ORR was 35.7% (90% CI, 15.3, 61.0), with five PRs. The DCR based on BOR was 71.4% (90% CI, 46.0, 89.6). The median PFS was 2.9 months (range, 1.3–19.2+) (Figure 1B). Nine of 14 patients (64.3%) experienced an event of PD (*n* = 7) or died (*n* = 2) during the study. The 6- and 12-month PFS rates were both 32.7% (90% CI, 13.3, 53.7). Among confirmed responders in the efficacy evaluable population, the median DOR was not estimable (90% CI, 2.6, NE). Median OS was 15.7 months (90% CI, 7.7, NE) (Figure 2B).

In the ibrutinib plus paclitaxel cohort, the median PFS was 4.1 months (range, 1.0–37.4+) (Figure 1C), which met the prespecified criteria for clinical significance. There were 56 PFS events, including 41 (72%) events of PD and 15 deaths (26%). PFS estimates at 6, 12, and 24 months were 30% (90% CI, 20.3, 39.9), 3.5% (90% CI, 0.9, 9.2), and 1.8% (90% CI, 0.2, 6.7), respectively. The best confirmed ORR was 26% (90% CI, 17.0, 37.6), with 2 CRs and 15 PRs. The DCR based on BOR was 66.7% (90% CI, 55.0, 77.0). Median OS was 8.2 months (90% CI, 6.2, 10.3) (Figure 2C). An efficacy summary is shown in Table 2.

### 3.4. Phase 1b Safety

Of the ten treated patients in phase 1b of the single-agent ibrutinib cohort, seven were DLT- evaluable and no DLTs were reported. Any-grade TEAEs were reported for 90% (*n* = 9/10) of patients; the most frequent TEAEs (≥4 patients) were decreased appetite (*n* = 6) and nausea and vomiting (*n* = 5 each). In the ibrutinib plus pembrolizumab cohort, 10 of 17 treated patients were DLT evaluable; no DLTs were reported. Any-grade TEAEs were reported for 94% of patients; the most frequent TEAEs (≥4 patients) were diarrhea and fatigue (*n* = 8 each); constipation (*n* = 7); hematuria, nausea, and arthralgia (*n* = 6 each); maculopapular rash (*n* = 5); and dizziness, stomatitis, and edema peripheral (*n* = 4 each). Of the 14 treated patients in phase 1b of the ibrutinib plus paclitaxel cohort, 10 were DLT evaluable, including four and six patients who received paclitaxel plus 560 mg and 840 mg ibrutinib, respectively. No DLTs were reported. All patients in phase 1b experienced an any-grade TEAEs; the most frequent TEAEs (>25% of patients) were diarrhea (86%), fatigue (57%), decreased appetite (64%), alopecia, constipation, and nausea (43% each), anemia (36%), and asthenia, hematuria, stomatitis, and vomiting (29% each).

### 3.5. Safety at RP2D

A safety summary for each cohort is shown in Table S2. The most common TEAEs of any-grade and grade ≥3 events for each cohort are listed in Table 3.

In the single-agent ibrutinib cohort, the median time on treatment was 1.4 months (range, 0.1–6.3). A total of 34 of 35 (97%) patients treated at the RP2D experienced a TEAE, with grade ≥3 TEAEs occurring in 66% (*n* = 23/35) of patients. Seven deaths occurred due to TEAEs or underlying UC (*n* = 7/35; 20%), none of which, including lower gastrointestinal hemorrhage, multiple organ dysfunction syndrome, sudden cardiac death, COVID-19 infection, Klebsiella infection, septic shock, and transitional cell carcinoma (*n* = 1 each), were deemed related to ibrutinib. For patients treated with ibrutinib plus pembrolizumab, the median time on treatment was 2.2 months (range, 0.1–19.9); 17 of 18 patients (94%) treated at the RP2D experienced a TEAE, with grade ≥3 TEAEs occurring in 72% (*n* = 13/18) of patients. Three deaths occurred due to TEAEs (*n* = 3/18; 17%) that included sudden death, cerebrovascular accident, and respiratory failure (*n* = 1 each). In the ibrutinib plus paclitaxel cohort, the median time on treatment was 2.5 months (range, 0.2–38). All 59 patients treated at the RP2D experienced a TEAE, with grade ≥3 TEAEs occurring in 81% (*n* = 48/59) of patients. Seven deaths occurred due to TEAEs or underlying UC (*n* = 7/59; 12%) and included general physical health deterioration (*n* = 3), transitional cell carcinoma (*n* = 2), Pneumocystis jirovecii pneumonia (*n* = 1), and urosepsis (*n* = 1).

In each cohort, the primary reason for discontinuation of ibrutinib was PD: 61% (ibrutinib plus paclitaxel), 60% (ibrutinib), and 33% (ibrutinib plus pembrolizumab) (Table 1).

### 3.6. Pharmacokinetics

Ibrutinib was rapidly absorbed, with a median time to a maximum concentration of 1.94 to 3.71 h across the three cohorts, regardless of the dose. Ibrutinib exposures at an RP2D dose of 840 mg in combination with paclitaxel appeared to be higher than with ibrutinib alone (1.7- and 1.8-fold higher mean Cmax and AUC0-24h, respectively). However, high inter-patient variability for exposure parameters was observed with values ≥90% for Cmax and AUC0-24h at the RP2D across all three cohorts. Paclitaxel Cmax ranged from 408 to 5690 ng/mL following administration of 59.3 to 83.1 mg/m^2^ paclitaxel with ibrutinib at the RP2D of 840 mg. A summary of pharmacokinetic parameters for ibrutinib is shown in Table S3.

### 3.7. Biomarkers

**Figure 3:**
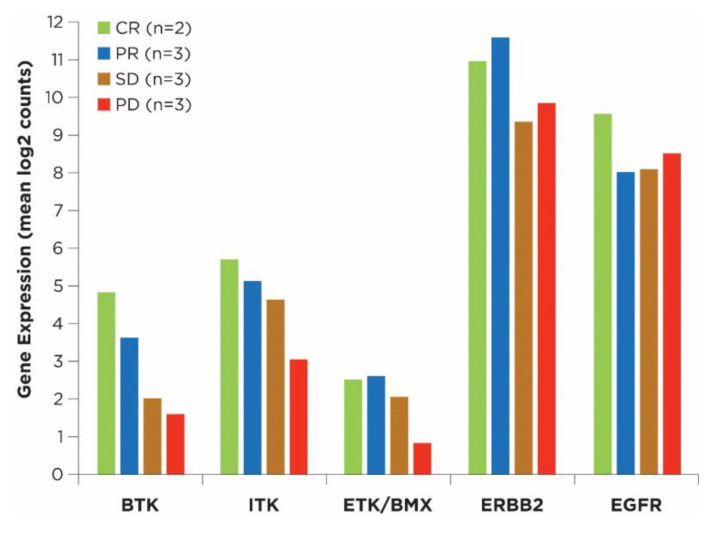
Relevant kinase expression in baseline tumor samples from the ibrutinib plus paclitaxel cohort. Included two patients from phase 1b who received ibrutinib 560 mg. Abbreviations: BTK, Bruton’s tyrosine kinase; CR, complete response; EGFR, epidermal growth factor receptor; ERBB2, ERB-B2 receptor tyrosine kinase 2; ETK/BMX, epithelial and endothelial tyrosine kinase/bone marrow X kinase; ITK, interleukin-2–inducible T-cell kinase; PD, progressive disease; PR, partial response; SD, stable disease.

Kinase expression was analyzed for a subset of patients receiving ibrutinib with paclitaxel (*n* = 11). Based on visual inspection and relative levels of expression versus depth of response, preliminary baseline tumor data showed a potential association of higher gene expression of BTK, ITK, and BMX with patients who had a treatment response, CR, PR, or stable disease versus patients with PD (Figure 3), though this trend was not significant.

In 27 pre-treatment tumor samples from patients receiving ibrutinib plus paclitaxel, subsequent assessments of CR, PR, or SD were significantly associated with higher baseline B- cell signature score (*p* = 2.9 × 10^−4^; average score in non-PD: 0.53; average score in PD: 0.19) and lower baseline peroxisome proliferator-activated receptor γ/retinoid X receptor α (PPARG/RXR) signature score (*p* = 2.1 × 10^−6^; average score in non-PD: 0.47; average score in PD: 0.88) compared to patients (Figure S1A). Two genes associated with histone-modifying enzymes, *KDM6A* and *KMT2D*, were mutated more significantly in responders (CR and PR) than in non-responders (SD and PD) (*p* = 0.03; odds ratio, 5.4) (Figure S1B). High baseline B-cell signatures and mutations in *KDM6A* or *KMT2D* appear to occur in non-overlapping subsets of responders (Figure S1C).

## 4. Discussion

In this multicenter, open-label, phase 1b/2 study, ibrutinib was found to have promising activity in combination with pembrolizumab or paclitaxel in patients with advanced UC. Single-agent ibrutinib, on the other hand, was found to have modest activity, and with a best confirmed ORR of 6.9%, the primary endpoint of the single-agent ibrutinib cohort was not met, although these data are helpful in understanding the contribution of components for the combination cohorts [15,16]. The 12-month OS rate of 38% suggests a limited value for single-agent ibrutinib in UC.

Although cross-trial comparisons are complicated by differences in study design and patient population, in the ibrutinib plus pembrolizumab cohort, the confirmed ORR of 35.7% and median OS of 15.7 months compares favorably with those reported with single-agent pembrolizumab in the second-line setting in an intention-to-treat population (21.1% ORR [95% CI, 16.4–26.5] and median OS 10.3 months) [17]. By contrast, the PFS for ibrutinib plus pembrolizumab was not significantly different compared with historic single-agent pembrolizumab data at 2.9 and 2.1 months, respectively. Interestingly, this apparently attenuated PFS effect is similar to findings from the KEYNOTE-045 trial, where median PFS in the pembrolizumab arm was inferior to that recorded in the chemotherapy arm (2.1 months [95% CI, 2.0–2.2] versus 3.3 months [95% CI, 2.3–3.5], respectively) [18], raising the possibility that PFS may not be the best endpoint for the evaluation of agents with immunomodulatory activity (such as with checkpoint inhibitors) in this particular clinical context. Because this cohort was terminated before reaching the stage 1 milestone of the Simon’s 2-stage design, the primary endpoint of ORR was not tested in the ibrutinib plus pembrolizumab cohort. Importantly, the early study termination was not because of safety or efficacy concerns but because of the sponsor’s decision. Given the small number of patients evaluated and the well-documented diversity of baseline disease parameters (five patients were treatment-naive with a PD-L1 score [combined positive score] of ≥10), which may influence outcomes with checkpoint inhibitors [6,19,20,21,22], a randomized trial comparing ibrutinib plus pembrolizumab with single-agent pembrolizumab would be necessary to further evaluate the promising initial signals of efficacy. Interestingly, in a randomized phase 2 study of pembrolizumab versus pembrolizumab plus acalabrutinib in patients with platinum-resistant metastatic UC, the addition of acalabrutinib did not improve the ORR, PFS, or OS when compared with pembrolizumab alone [23]. In contrast to ibrutinib, acalabrutinib does not inhibit ITK [12,24], which may explain the efficacy difference observed in UC between these two BTK inhibitors. Although the contribution of ITK inhibition in immune response in UC is unclear [25] and the ibrutinib and pembrolizumab data were collected in a single, small cohort of patients, further work, including biomarker analysis, may be valuable in understanding the patient population that could benefit from a BTK inhibitor and pembrolizumab combination [23].

The median PFS of 4.1 months in patients being treated with ibrutinib plus paclitaxel met the prespecified criteria for statistical significance. At 26%, the confirmed ORR of ibrutinib plus paclitaxel is higher than historical data for single-agent taxanes and is substantially higher than that of ibrutinib monotherapy; however, the median PFS and OS of 4.1 and 8.2 months, respectively, are comparable to those observed in single-agent taxane trials [26,27,28,29,30]. Biomarker analyses suggest that high B-cell content and mutations in epigenetic regulators *KDM6A* and *KMT2D* are predictive of response in a simple cross-validated model. These initial data are limited by the small number of patient samples with a finite number of responders and non-responders. Despite these limitations, there appear to be relevant signatures that are hypothesis-generating. The findings require validation on an extended sample set or in additional clinical studies.

## 5. Conclusions

The initial observation of response, the manageable safety profile seen across all three cohorts, and the high levels of relative dose delivery of both ibrutinib and partner agents support the feasibility of the regimens studied, thereby warranting consideration of further evaluation of these agents in UC. Considering the evolving treatment landscape of UC, with new agents being approved [31,32,33,34] and checkpoint inhibitors being used as maintenance therapy in first-line metastatic UC [6], in addition to checkpoint inhibitors gaining approval in earlier lines of UC therapy [35], the patient population should be selected thoughtfully by identifying where an ibrutinib combination regimen may provide the greatest benefit. While significant treatment advancements have recently been made, UC remains a devastating disease with high recurrence and high progression rates for which durable treatment options are needed.

## Figures and Tables

**Table 1:** Baseline patient characteristics and patient disposition for the RP2D populations. ^a^

	Ibrutinib 840 mg *N* = 35 ^b^	Ibrutinib 560 mg + Pembrolizumab *N* = 18	Ibrutinib 840 mg + Paclitaxel *N* = 59
**Median age (range), years**	71 (52–88)	70 (52–84)	68 (48–90)
>65 years, *n* (%)	24 (69)	12 (67)	36 (61)
**Male, *n* (%)**	26 (74)	13 (72)	51 (86)
**Race, *n* (%)**			
White	30 (86)	16 (89)	47 (80)
Black or African American	0	1 (6)	0
Asian	5 (14)	0	12 (20)
American Indian/Alaska Native	0	1 (6)	0
**ECOG performance status, *n* (%)**			
0	8 (23)	7 (39)	9 (15)
1	27 (77)	11 (61)	50 (85)
**Time from initial diagnosis to start of treatment, median (range), months**	38 (15–129)	17 (2–85)	20 (5–161)
**Metastatic sites of disease, *n* (%)**			
0	0	1 (6)	1 (2)
1	9 (26)	4 (22)	13 (22)
2	15 (43)	7 (39)	24 (41)
>2	11 (31)	6 (33)	21 (36)
**Sites of metastasis, *n* (%)**			
With metastases	34 (97)	17 (94)	56 (95)
Bone	11 (31)	7 (39)	14 (24)
Liver	13 (37)	5 (28)	12 (20)
Lung	16 (46)	10 (56)	25 (42)
Lymph node	25 (71)	10 (56)	46 (78)
Peritoneal	6 (17)	3 (17)	5 (8)
**Number of prior regimens, *n* (%)**			
0	N/A	5 (28)	N/A
1	5 (14)	11 (61)	28 (47)
2	30 (86)	2 (11)	31 (53)
**Treatment duration, ibrutinib, median (range), months**	1.4 (0.1–6.3)	2.2 (0.1–19.9)	2.3 (0.2–38.0)
**Treatment duration of partner drug, median (range), months**	N/A	1.7 (0.0–19.4)	1.6 (0.0–5.6)
**Ibrutinib treatment disposition, n (%)**			
Still on treatment	0	0	0
Discontinued treatment	35 (100)	18 (100)	59 (100)
**Primary reason for ibrutinib discontinuation, *n* (%)**			
Disease progression	21 (60)	6 (33)	36 (61)
Clinical deterioration	4 (11)	0	4 (7)
Adverse events unrelated to PD or TCC	5 (14)	3 (17)	9 (15)
Death	2 (6)	3 (17)	1 (2)
Withdrawal of consent	3 (9)	4 (22)	7 (12)
Investigator decision	0	0	2 (3)
Study terminated by sponsor	0	2 (11)	0
**Companion drug treatment disposition, *n* (%)**			
Still on treatment	N/A	0	0
Discontinued treatment	N/A	18 (100)	59 (100)
**Primary reason for discontinuation of companion drug, *n* (%)**			
Disease progression	N/A	6 (33)	27 (46)
Clinical deterioration	N/A	0	1 (2)
Adverse events unrelated to PD	N/A	1 (6)	24 (41)
Death	N/A	3 (17)	0
Withdrawal of consent	N/A	6 (33)	7 (12)
Investigator decision	N/A	0	0
Study terminated by sponsor	N/A	2 (11)	0

Abbreviations: ECOG, Eastern Cooperative Oncology Group; N/A, not applicable; PD, progressive disease; RP2D, recommended phase 2 dose; TCC, transition cell carcinoma. ^a^ Data cutoff date: 4 October 2021. ^b^ Parameters noted as “N/A” in the single-agent ibrutinib cohort are not applicable either because of eligibility criteria or the lack of a companion drug.

**Table 2:** Efficacy summary (in phase 1b/2 efficacy-evaluable population).

	Ibrutinib 840 mg *N* = 29	Ibrutinib 560 mg + Pembrolizumab *N* = 14	Ibrutinib 840 mg + Paclitaxel *N* = 57
**Median PFS (range), months**	1.6 (0.9–8.2)	2.9 (1.3–19.2+)	4.1 (1.0–37.4+)
**Best confirmed ORR, % (90% CI)**	6.9 (1.2, 20.2)	35.7 (15.3, 61.0)	26.3 (17.0, 37.6)
**Best overall response, *n* (%)**			
CR	0	0	2 (3.5)
PR	2 (6.9)	5 (35.7)	13 (22.8)
SD	12 (41.4)	5 (35.7)	23 (40.4)
PD	15 (51.7)	4 (28.6)	12 (21.1)
Not evaluable	0	0	1 (1.8)
Unknown/missing	0	0	6 (10.5)
**DCR, % (90% CI)**	48.3 (32.0, 64.8)	71.4 (46.0, 89.6)	66.7 (55.0, 77.0)
**Median OS, (90% CI), months**	8.2 (4.4, NE)	15.7 (7.7, NE)	8.2 (6.2, 10.3)

Abbreviations: CR, complete response; DCR, disease control rate; ORR, overall response rate; OS, overall survival; NE, not evaluable; PD, progressive disease; PFS, progression-free survival; PR, partial response; SD, stable disease.

**Table 3:** Most common treatment-emergent adverse events (TEAEs) of any-grade and grade ≥3 events for each study cohort.

	Ibrutinib 840 mg *N* = 35		Ibrutinib 560 mg + Pembrolizumab *N* = 18		Ibrutinib 840 mg + Paclitaxel *N* = 59	
TEAE ^a^	Any Grade (≥20%)	Grade ≥3 (≥10%)	Any Grade (≥20%)	Grade ≥3 (≥10%)	Any Grade (≥20%)	Grade ≥3 (≥10%)
Diarrhea	10 (29)	2 (6)	8 (44)	0	44 (75)	6 (10)
Asthenia	13 (37)	1 (3)			31 (53)	13 (22)
Decreased appetite	12 (34)	0			25 (42)	2 (3)
Anemia	7 (20)	2 (6)			24 (41)	10 (17)
Nausea	16 (46)	1 (3)	6 (33)	0	23 (39)	1 (2)
Fatigue	10 (29)	2 (6)	8 (44)	1 (6)	19 (32)	4 (7)
Constipation	9 (26)	0	7 (39)	0	19 (32)	0
Alopecia					18 (31)	1 (2)
Stomatitis			4 (22)	0	17 (29)	1 (2)
Vomiting	10 (29)	0			17 (29)	1 (2)
Peripheral sensory neuropathy					16 (27)	6 (10)
Edema peripheral			4 (22)	1 (6)	15 (25)	0
Urinary tract infection	7 (20)	2 (6)			13 (22)	7 (12)
Arthralgia			6 (33)	0	13 (22)	1 (2)
Hematuria			6 (33)	1 (6)	14 (24)	1 (2)
Dizziness	9 (26)	0	4 (22)	0		
Rash maculopapular			5 (28)	1 (6)		

Abbreviation: TEAE, treatment-emergent adverse event. ^a^ Adverse events listed in descending order based on any grade incidence in the ibrutinib plus paclitaxel cohort. Gray cells indicate the TEAE was not observed in the cohort.

## Data Availability

Requests for access to individual participant data from clinical studies conducted by Pharmacyclics LLC, an AbbVie Company, can be submitted through Yale Open Data Access (YODA) Project site at http://yoda.yale.edu.

## Supplementary Materials

The following supporting information can be downloaded at: https://www.mdpi.com/article/10.3390/cancers15112978/s1, Figure S1: Biomarker analyses of baseline tumor samples from the ibrutinib plus paclitaxel cohort; Table S1: Representativeness of study participants; Table S2: Safety summary in all-treated phase 1/b patients; Table S3: Mean (SD) pharmacokinetic parameters of ibrutinib in plasma following once-daily oral dosing of ibrutinib in patients with UC.
