# Supplementary material for: Results from a Phase 1b/2 Study of Ibrutinib Combination Therapy in Advanced Urothelial Carcinoma
Source: Cancers (Basel). 2023 May 30;15(11):2978. doi: 10.3390/cancers15112978 (PMC10251876; doi:10.3390/cancers15112978)
Supplement: Supplementary file 1 [file cancers-15-02978-s001.zip › cancers-2346876-supplementary.docx]

**Supplementary Information**

**Results From a Phase 1b/2 Study of Ibrutinib Combination Therapy in Advanced Urothelial Carcinoma**

Nataliya Mar^*^, Yousef Zakharia^*^, Alejandro Falcon, Rafael Morales-Barrera, Begona Mellado, Ignacio Duran, Do-Youn Oh, Stephen K. Williamson, Pablo Gajate, Hendrik-Tobias Arkenau, Robert J. Jones, Min Yuen Teo, Hillary M. Peltier, Elizabeth Chong, Harisha Atluri, James P. Dean, and Daniel Castellano

*Co-first authorship, equal contribution

**Table of Contents**

**Supplementary Methods.** Study design and dose-limiting toxicities 2

**Supplementary Methods.** Response: Target and non-target lesions 3

**Supplementary Methods.** Maximum tolerated dose 3

**Supplementary Methods.** Dose adjustment guidelines 3

**Supplementary Methods.** Efficacy in phase 1b 4

**Supplementary Methods.** RNA- and DNA-seq analyses 4

**Supplementary Table S1.** Representativeness of study participants. 6

**Supplementary Table S2.** Safety summary in all-treated phase 1/b patients. 8

**Supplementary Table S3.** Mean (SD) pharmacokinetic parameters of ibrutinib in plasma following once-daily oral dosing of ibrutinib in patients with UC. 9

**Supplementary Figure S1**. Biomarker analyses of baseline tumor samples from the ibrutinib plus paclitaxel cohort: B cell-differentiated vs *KDM6A* or *KMT2D* responders…………....10

**Supplementary Methods**

Patients were followed every 3 months after study treatment ended.

*Study design and dose-limiting toxicities*

A 6 + 3 dose de-escalation design was used for patients receiving ibrutinib plus pembrolizumab to evaluate dose-limiting toxicities (DLTs) and determine the recommended phase 2 dose (RP2D). A DLT was defined as any grade 3 or higher nonhematologic or grade 4 hematologic adverse event (AE) possibly related to either ibrutinib or the drug combination that occurred during the DLT-observation period. If 2 patients within the initial cohort of 6 patients experienced a DLT, an additional 3 patients were enrolled at the same dose level. If ≥3 of the 6 patients experienced a DLT, dose de-escalation occurred. If incidence of DLTs during the observation period was <33.3%, the dose level was defined as the RP2D. The DLT observation period was 21 days following the initiation of combination therapy at the start of cycle 1. Stage 1 of the Simon’s 2-stage design had a target enrollment of at least 31 patients, including any from phase 1b who were treated at the RP2D. For patients receiving ibrutinib plus paclitaxel, phase 1b followed a 3 + 3 + 3 design. The 3 + 3 + 3 design refers to a procedure in which DLTs are assessed in the first 3 evaluable patients at each dose level by a safety review committee, which could be expanded to 6 patients if 1 of the initial 3 experienced a DLT or 9 patients if a DLT was observed in 2 of the 6 patients. The phase 2 portion enrolled patients until at least 55 patients were treated, including any from phase 1b who were treated at the RP2D.

*Response*

*Target lesions*

Complete response (CR) was defined as disappearance of all target lesions. Any pathological lymph nodes (whether target or nontarget) must have a reduction in the short axis to <10 mm. Partial response (PR) was defined as at least a 30% decrease in the sum of diameters of target lesions, taking as reference the baseline sum diameters.

*Non-target lesions*

CR was defined as disappearance of all nontarget lesions and normalization of tumor marker level. All pathological lymph nodes must be non-pathological in size (short axis to <10 mm).

*Maximum tolerated dose*

In a previous phase 1 study in which patients received up to 12.5 mg/kg per day (1400 mg/day) of ibrutinib, no maximum tolerated dose was reached (Advani RH et al. *J Clin Oncol.* 2013;31:1:88‒94).

*Dose adjustment guidelines*

Dose modifications were not permitted in phase 1b in the absence of a DLT. However, the dose of ibrutinib was modified in cases of the following: grade 4 neutropenia (absolute neutrophil count <500/μL) for >7 days; grade 3 thrombocytopenia (platelets <50,000/μL) with clinically significant bleeding events; grade 4 thrombocytopenia (platelets <25,000/μL); grade 3 or 4 nausea, vomiting, or diarrhea if persistent despite optimal antiemetic and/or antidiarrheal therapy; and any other grade 4 or unmanageable grade 3 toxicity.

*Efficacy in phase 1b*

In the single-agent ibrutinib cohort, the efficacy-evaluable population included 8 patients who received ibrutinib 840 mg daily. The best confirmed overall response rate (ORR) was 12.5% (90% CI, 0.6, 47.1), including 1 PR (12.5%). The disease control rate (DCR) based on best overall response (BOR) was 37.5% (90% CI, 11.1, 71.1). With ibrutinib plus pembrolizumab, the efficacy-evaluable population in phase 1b included patients who received ibrutinib 560 mg daily plus pembrolizumab (*n* = 13). The best confirmed ORR was 38.5% (90% CI, 16.6, 64.5), including 5 PRs. The DCR based on BOR was 76.9% (90% CI, 50.5, 93.4). In the ibrutinib plus paclitaxel cohort, all 14 patients in phase 1b were included in the efficacy-evaluable population. The best confirmed ORR was 29% (90% CI, 10.4, 54.0), including 2 CRs and 2 PRs (14% each). The DCR based on BOR was 57% (90% CI, 32.5, 79.4).

*RNA- and DNA-seq analyses*

Raw RNA-seq Personalis FASTQ files were quality and adapter-trimmed and aligned to the hg19 human reference genome; a gene by sample count matrix was generated. Quality control metrics, including unique alignment percentage, total informative and non-zero gene counts, and rRNA contamination rates were considered. Differential expression was evaluated using the DESeq2 method. Between- and within-lane normalizations were applied when conducting signature analyses (EDASeq). Single-sample geneset enrichment scores were calculated using yaGST method. The outcome association was evaluated via student’s t-test (ORR) or Cox proportional hazards model (PFS).

For mutation analyses, Personalis FASTQ files were aligned to the hg38 human reference genome using Sentieon Genomics Tools implementation of Burrows-Wheeler alignment- Maximal Exact Match (BWA-MEM). Binary alignment map files (BAMs) were sorted, de-duplicated, and base recalibrated according to the recommended commands. Matched tumor-normal variant calling was performed with Sentieon's TNScope using an ACE ImmuneID NEXT Normal panel variant call format (VCF). Quality somatic variants were retained by filtering such that TNScope = PASS, Varscan2 FPfilter = PASS, and variants were not present in the normal panel VCF. Variants occurring in COSMIC (catalog of somatic mutations in cancer) were white-listed.

**Table S1.** Representativeness of study participants.

| **Cancer type** | **Urothelial carcinoma** |
| --- | --- |
| **Considerations related to:** | |
| Sex | Bladder cancer, urothelial carcinoma being one of the most common types, is four times more common in men than women (1). |
| Age | Most bladder cancer diagnoses are made in patients who are aged ≥55 years; in the United States, 80% of diagnoses are in patients aged >65 years. The average age at diagnosis in the United States is 73 years (1). |
| Race/ethnicity | Results from a study investigating racial differences in bladder cancer (both urothelial and squamous types) suggested that survival of Black and Native American/Alaska Native patients is lower than that of other populations. Asian and Pacific Islander populations had the highest survival in this study (2). |
| Geography | The highest rates of bladder cancer are found in countries in Southern and Western Europe and North America. Greece and Lebanon have the highest rates of bladder cancer in men and women, respectively (1). |
| Other considerations | The main risk factor for bladder cancer is exposure to tobacco smoke. Exposure to environmental and occupational chemicals, particularly those associated with industrial production of dyes, paint, rubber, metal, or petroleum products is also a risk factor (1). |
| Overall representativeness of this study | The age distribution of our study is similar to the average age distribution of bladder cancer in the literature, with a median age of 68 to 71 years with two-thirds of patients aged >65 years.  The majority of participants in this study were male, which aligns with the literature. |

1. Saginala K, Barsouk A, Aluru JS, Rawla P, Padala SA, Barsouk A. Epidemiology of bladder cancer. Med Sci 2020;8(1):15.
2. Fang W, Yang Z-Y, Chen T-Y, Shen X-F, Zhang C. J Transl Med 2020;18:145.

**Table S2.** Safety summary in all-treated phase 1/b patients.

| ***n* (%)** | **Ibrutinib 840 mg**  ***N* = 35** | **Ibrutinib 560 mg + pembrolizumab**  ***N* = 18** | **Ibrutinib 840 mg + paclitaxel**  ***N* = 59** |
| --- | --- | --- | --- |
| **TEAE (any grade)** | 34 (97) | 17 (94) | 59 (100) |
| Grade ≥3 TEAE | 23 (66) | 13 (72) | 48 (81) |
| **Patients with TEAEs leading to discontinuation of study treatment^a^** | 7 (20) | 5 (28) | 25 (42) |
| Ibrutinib only | 7 (20) | 3 (17) | 1 (2) |
| Companion drug only | N/A | 0 | 15 (25) |
| Both ibrutinib and companion drug | N/A | 2 (11) | 9 (15) |

Abbreviations: TEAE, treatment-emergent adverse event.

^a^Includes adverse events with action taken as study treatment permanently withdrawn.

**Table S3.** Mean (SD) pharmacokinetic parameters of ibrutinib in plasma following once-daily oral dosing of ibrutinib in patients with UC.

| **Treatment** | **Ibrutinib dose**  **(mg)** | ***N*** | **C_max_**  **(ng/mL)** | **t_max_^a^**  **(h)** | **AUC_0-24h_ (ng·h/mL)** | **t_1/2,term_**  **(h)** |
| --- | --- | --- | --- | --- | --- | --- |
| Single-agent ibrutinib | 840 | 24 | 246 (201) | 2.00 (1.00, 6.00) | 1957^d^ (1609) | 6.84^e^ (1.90) |
| Ibrutinib + pembrolizumab^b^ | 560 | 10 | 256 (158) | 1.94 (1.08, 3.87) | 1334^f^ (767) | 6.35^g^ (1.26) |
| Ibrutinib + paclitaxel^c^ | 560  840 | 10  35 | 260 (287)  424 (319) | 3.71 (1.00, 6.00)  4.00 (0.833, 6.00) | 2194 (3023)  3503^h^ (2613) | 7.24^i^ (2.36)  4.31^f^ (1.95) |

Abbreviations: AUC_0-24h_, area under the concentration-time curve from time 0 to 24 hours; C_max_, observed maximum concentration; SD, standard deviation; t_1/2, term_, terminal elimination half-life; t_max_, time to C_max_; UC, urothelial carcinoma.

^a^Median (minimum, maximum) presented for t_max_.

^b^Patients were to be dosed with 200 mg intravenous every 3 weeks.

^c^Patients were to be dosed with paclitaxel 80 mg/m^2^ once weekly, in continual weekly cycles.

^d^*N* = 22.

^e^*N* = 10.

^f^*N* = 7.

^g^*N* = 3.

^h^*N* = 34.

^i^*N* = 2

**Figure S1.** Biomarker analyses of baseline tumor samples from the ibrutinib plus paclitaxel cohort^a^: (**A**) Boxplots showing B-cell signature score (Saleh et al. *Cancer Res.* 2017*;*77:4673) and PPARG/RXR signature score (Korpal et al. *Nat Commun.* 2017;8:103) between response groups. (**B**) Two genes associated with histone modifying enzymes, *KDM6A* and *KMT2D,* were mutated more significantly in responders (CR and PR) than in non-responders (SD and PD) (**C**) Heatmap of gene mutations and expression signatures. Responders with high B-cell signatures (red box) are different from responders with *KDM6A* or *KMT2D* mutations (purple boxes) Abbreviations: CR, complete response; PD, progressive disease; PR, partial response; PPARG, peroxisome proliferator activated receptor γ; RXR, retinoid X receptor α; SD, stable disease.

A


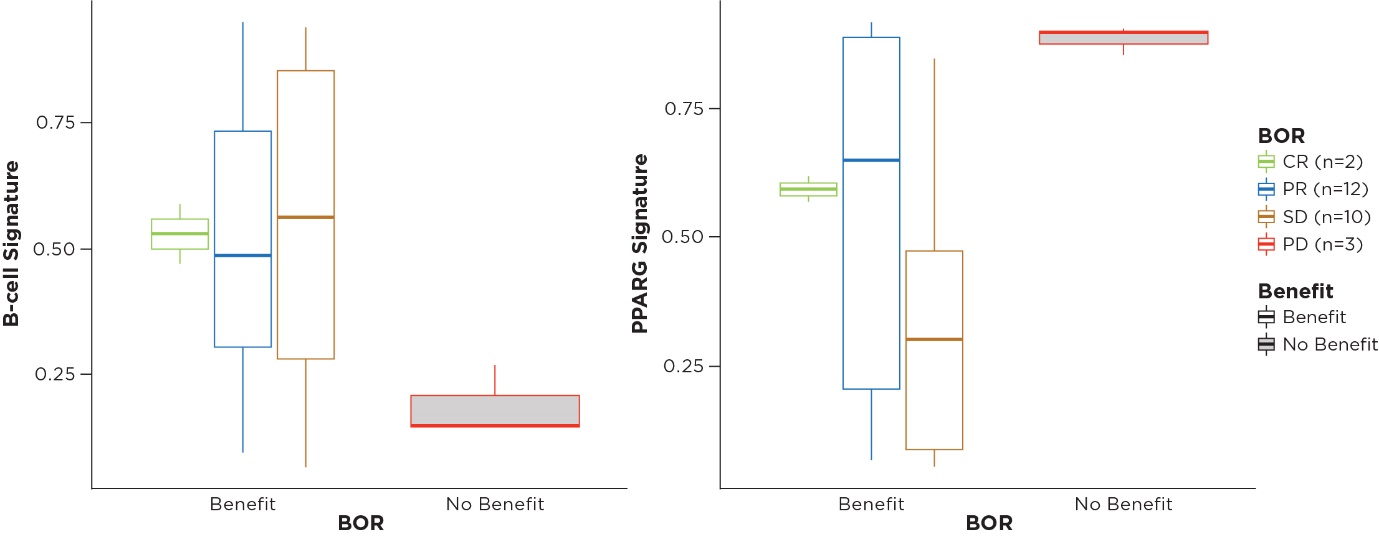


B


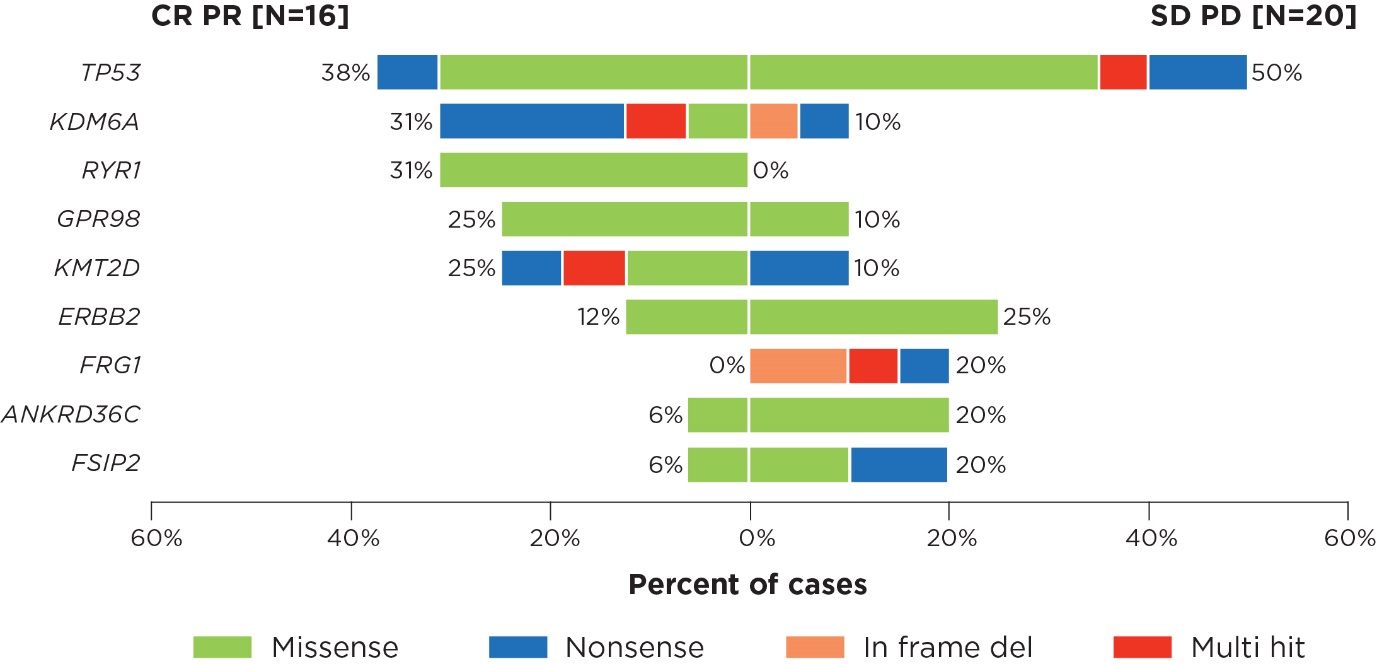


C


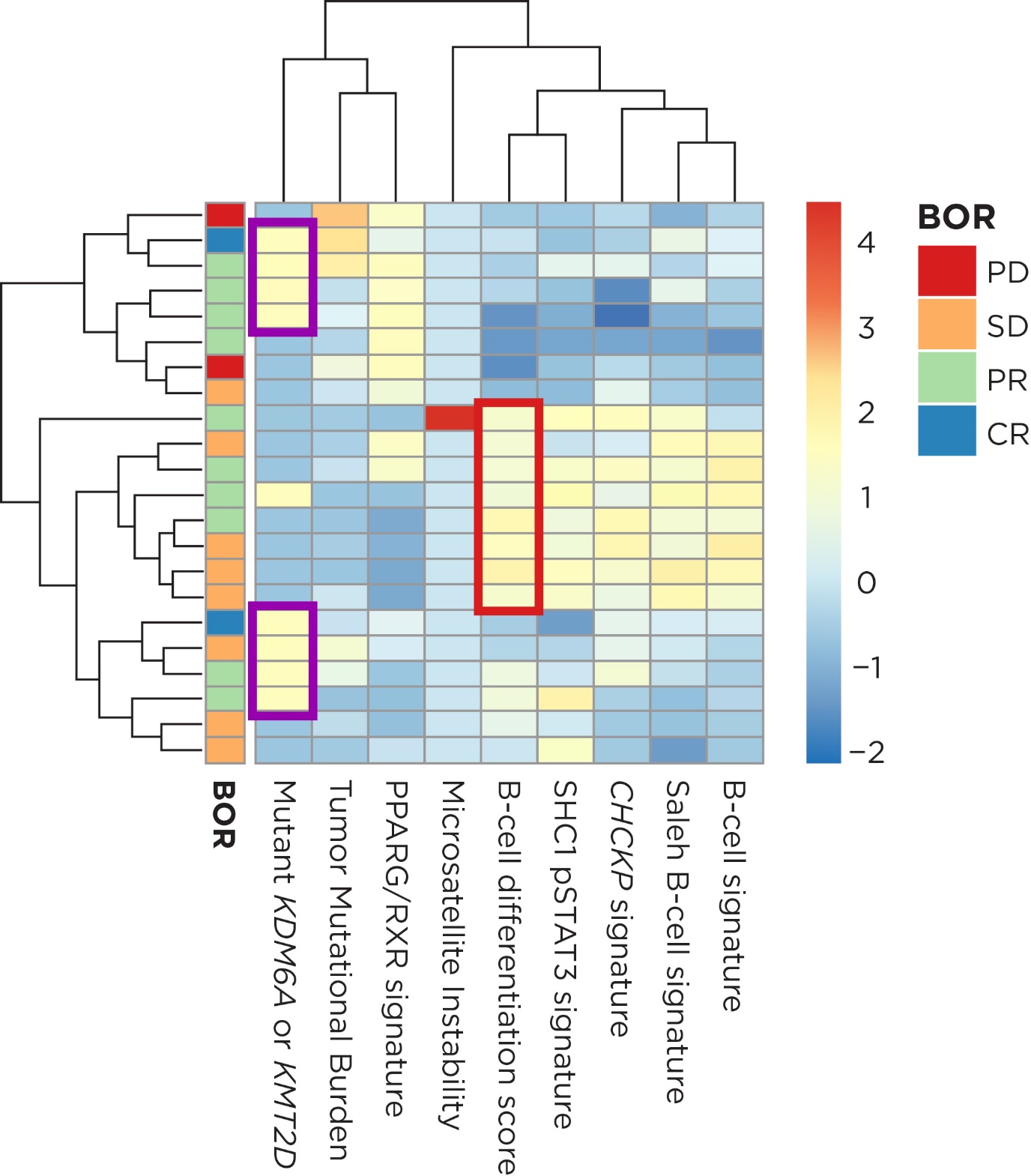


^a^ One patient sample had low values for all QC metrics and clustered distinctly from the rest of the samples, therefore this RNAseq sample was removed from the rest of the biomarker analysis.
